# Genome-wide association analysis of metabolic syndrome quantitative traits in the GENNID multiethnic family study

**DOI:** 10.1186/s13098-021-00670-3

**Published:** 2021-06-01

**Authors:** Jia Y. Wan, Deborah L. Goodman, Emileigh L. Willems, Alexis R. Freedland, Trina M. Norden-Krichmar, Stephanie A. Santorico, Karen L. Edwards, American Diabetes GENNID Study Group, Eric Boerwinkle, John Buse, Ralph DeFronzo, David Ehrmann, Steven C. Elbein, Wilfred Fujimoto, Steven E. Kahn, Craig L. Hanis, Richard A. Mulivor, Jeanne C. Beck, Jill Norris, M. Alan Permutt, Philip Behn, Leslie Raffel, David C. Robbins

**Affiliations:** 1 Department of Epidemiology and Biostatistics, Program in Public Health, University of California, 635 E. Peltason Dr, Mail Code: 7550, Irvine, CA 92697 USA; 2 Department of Mathematical and Statistical Sciences, University of Colorado, Denver, CO USA; 3 Human Medical Genetics and Genomics Program, University of Colorado, Denver, CO USA; 4 Department of Biostatistics & Informatics, University of Colorado, Denver, CO USA; 5 Division of Biomedical Informatics & Personalized Medicine, University of Colorado School of Medicine, Aurora, CO USA

**Keywords:** Metabolic syndrome, Genetic epidemiology, Family studies, Quantitative trait loci, Linkage

## Abstract

**Background:**

To identify genetic associations of quantitative metabolic syndrome (MetS) traits and characterize heterogeneity across ethnic groups.

**Methods:**

Data was collected from GENetics of Noninsulin dependent Diabetes Mellitus (GENNID), a multiethnic resource of Type 2 diabetic families and included 1520 subjects in 259 African-American, European-American, Japanese-Americans, and Mexican-American families. We focused on eight MetS traits: weight, waist circumference, systolic and diastolic blood pressure, high-density lipoprotein, triglycerides, fasting glucose, and insulin. Using genotyped and imputed data from Illumina’s Multiethnic array, we conducted genome-wide association analyses with linear mixed models for all ethnicities, except for the smaller Japanese-American group, where we used additive genetic models with gene-dropping.

**Results:**

Findings included ethnic-specific genetic associations and heterogeneity across ethnicities. Most significant associations were outside our candidate linkage regions and were coincident within a gene or intergenic region, with two exceptions in European-American families: (a) within previously identified linkage region on chromosome 2, two significant *GLI2-TFCP2L1* associations with weight, and (b) one chromosome 11 variant near *CADM1-LINC00900* with pleiotropic blood pressure effects.

**Conclusions:**

This multiethnic family study found genetic heterogeneity and coincident associations (with one case of pleiotropy), highlighting the importance of including diverse populations in genetic research and illustrating the complex genetic architecture underlying MetS.

**Supplementary Information:**

The online version contains supplementary material available at 10.1186/s13098-021-00670-3.

## Background

Metabolic syndrome (MetS) is a common, complex condition characterized by hyperlipidemia, hypertension, hyperglycemia, and excess abdominal fat [1–3]. The National Cholesterol Education Program’s Adult Treatment Panel (NCEP ATP) III criteria [3], typically used in the United States for clinical diagnosis, defines MetS as the presence of at least three of five risk factors: elevated systolic and/or diastolic blood pressure (SBP, DBP), elevated triglycerides (TG), decreased high density lipoprotein (HDL)-cholesterol, elevated fasting glucose, and abdominal obesity [1, 3]. Due to the clustering of these characteristics [4, 5], individuals with MetS are at risk for cardiovascular and metabolic diseases such as stroke and diabetes [6–10]. Moreover, in several US-based studies of families [11–15], MetS quantitative and multivariate factor traits are highly heritable with about half of the variation between subjects explained by genetics in families of European descent [14, 15] and particularly for obesity and lipid-related traits in families of African Americans [12, 14], Mexican Americans [13] and Japanese Americans [11]. Family-based studies have been a primary approach for identifying genetic influences on a range of disease and still offer many advantages [16, 17] including being robust to confounding due to underlying population structure and phenotype model misspecifications, using pedigree structures and information on related individuals to detect genotyping errors [16], and having more power to detect rare variants [16, 17].

Candidate gene [18–23] and genome-wide association studies (GWAS) [18, 24–27] have already generated a number of candidate genes and variants possibly associated with MetS. However, the number of variants is still growing [8], particularly in the Asian population [28]. Nonetheless, many questions still remain about the underlying genetic architecture of MetS. For example, are the genetic influences the same regardless of which NCEP traits cluster within an individual? Accumulating evidence suggests that the specific combination of traits may matter and could explain the large number of variants associated with MetS [29, 30]. Several obesity-related loci have been shown to be associated with different MetS traits [8, 31]. For example, obesity, high TG, high fasting insulin, and low HDL are associated with *MIP1*, *MC4R*, and *PRKD1*, yet when these same traits are combined with hypertension, they are associated with *FTO* and *TMEM18* [8].

Results from our previous studies suggest differences in the clustering due to the underlying genetics of MetS traits by ethnicity [32–34]. For example, while a significant genetic correlation between weight and waist is present in African American (AA), European American (EA), Japanese American (JA) and Mexican American (MA) families [32, 34], the genetic correlation between high systolic blood pressure (SBP) and diastolic blood pressure (DBP) is seen only in AA, EA, and MA families [34]. The significant genetic correlation of lipids (TG and HDL) has been shown to be characteristic among EA and JA families [32, 34]. These differences in clustering patterns may be driven by different sets of underlying genetic influences and could explain the large number of genetic variants and genes associated with MetS.

Previously, family-based genetic linkage analyses nominated chromosomal regions with putative causal variants for individual and multivariate MetS traits. Results indicated several high priority linkage regions, including a region on chromosome 2 for EA [32, 33] and AA families [35, 36] and a linkage region containing *ADIPOQ* on chromosome 3 among MA families [33]. These candidate linkage regions are large (between 150 and 540 Mbp), with multiple traits mapping to these regions and evidence for heterogeneity across ethnic groups [33]. A more in-depth evaluation of these regions to determine if linkage is due to pleiotropy or co-incident linkage/association, along with a broader focus on understanding if different trait clustering contributes to heterogeneity is needed. We used the GENetics of NonInsulin-dependent Diabetes mellitus (GENNID) resource [37], a multiethnic study of families with type 2 diabetes (T2D), and a GWAS approach to identify quantitative trait nucleotides (QTNs) with possible pleiotropic or coincident effects and to examine evidence for heterogeneity in genetic association findings for MetS traits across ethnic groups.

## Methods

### Study subjects

GENNID is an American Diabetes Association (ADA) resource of genetic, questionnaire, and laboratory data from multiplex, ethnically diverse AA, EA, JA and MA families with T2D, diagnosed using the National Diabetes Data Group criteria [38]. In this cross-sectional study from 1993 to 1997, T2D families were ascertained in two phases across multiple centers in the United States [37]. Phase 1 focused primarily on larger, multi-generational data collection of families with at least two T2D affected siblings in addition to at least three first-degree relatives. Phase 2 ascertained sibling pairs and nuclear families with at least two T2D affected siblings, and if at most one parent was ascertained, then data was collected on at least two additional siblings. AA, EA, and MA families were collected in both phases while JA families were only collected in Phase 1 [37, 39]. This study used all available data except for the Phase 2 EA data (N = 371 subjects) which were not yet genotyped. Self-identified race, family and medical histories, anthropometric and lab measurements were obtained from participants. Specifically, we focused on eight MetS-related, quantitative traits (i.e., HDL, TG, SBP, DBP, fasting insulin, fasting glucose, weight, and waist circumference) defined from anthropomorphic and lab measurements. Pedigree relationships, age, sex, and diabetes status were obtained from the data collection and questionnaires.

### Genotying and imputation

Previously, using microsatellite markers, linkage analyses identified candidate regions for multivariate MetS traits as described in Edwards et al. [32]. For this study, the Northwest Genomics Center (NWGC) performed genome-wide genotyping using Illumina’s Infininium LCG genotyping assay on the Multiethnic Global beadchip (v1.0, genome build 37). DNA samples were normalized using a PerkinElmer Janus Workstation and then genotyped. We performed quality control (QC) of genotype data separately for each ethnic group [40]. Genetic imputation was then performed by first phasing each ethnic group’s QC’ed genotypes using Eagle2 software via the Sanger Imputation Service [41] with the corresponding reference panels for each ethnic group: 1000 Genomes Phase 3 [42] reference panel (for AAs, MAs, JAs) and the Haplotype Reference Consortium (HRC) [41] reference panel (for EAs). DuoHMM [43] was used to correct haplotype phasing switch errors based on pedigree relatedness. The final step for imputation was then performed using Minimac3 via the Michigan Imputation Server [44] with the HRC reference panel.

### Statistical analysis

We performed genome-wide association testing across all imputed and genotyped QTNs. Quantitative traits with non-normal distributions were transformed in order to satisfy normality assumptions. The skewed distributions of HDL, TG, waist, and insulin measures were log-transformed, whereas a rank-based inverse normal transformation resulted in approximately normal distributions for weight, DBP, SBP, and fasting glucose. For AAs, EAs, and MAs, linear mixed models were used in Genome-wide Complex Trait Analysis (GCTA) software [45] with the kinship coefficient matrix empirically estimated by LD-adjusted kinships (LDAK) software [46]. However, due to asymptotic concerns with a smaller JA sample, association testing was performed using gene-dropping [40]. Univariate association analyses were adjusted by age, sex, and self-reported diabetes status. Association results with P ≤ 5 × 10^–8^ were genome-wide significant and with P between 5 × 10^–8^ and 10^–6^ (i.e., 5 × 10^–8^ < P ≤ 10^–6^) were suggestive of association. Additionally, after a Bonferroni correction for the testing of 8 traits, we also identified highly significant QTNs with P ≤ 6.25 × 10^–9^ [i.e., (5 × 10^–8^)/8]. Moreover, the *I*^*2*^ metric [47] was used to assess the degree of heterogeneity across ethnic groups. Using the METAL software [48], *I*^2^ was calculated as the percentage of variance that is due to heterogeneity of effect size (β) estimates across ethnic groups. *I*^2^ values of 0 indicate no heterogeneity of effect sizes across ethnic groups; values over 75% and up to 100% indicate considerable heterogeneity [49]. *I*^2^ values were not calculated for QTNs present in only one ethnic group (i.e., when the QTNs were monomorphic or were filtered out during QC in the other ethnic groups). The R program [50] was also used for statistical analysis, programming, and plotting. Circular Manhattan plots were made using the CMplot R package [51].

### Functional and regulatory annotation

Finally, evidence of biological function was characterized by annotating significant QTNs and any QTNs in linkage disequilibrium (LD). Specifically, we used ANNOtate VARiation (ANNOVAR) software [52] to annotate significant QTNs with five different integrative annotations and their corresponding thresholds. In particular, based on support vector machine (SVM) supervised learning, Combined Annotation Dependent Depletion (CADD v1.3) [53] phred-scaled scores of at least 10 denoted deleterious variants belonging in the top 10%. Additionally, obtained from random forest methods, Genome Wide Annotation of VAriants (GWAVA) annotation tool [54], Training Stress Scores (TSS) of at least 0.40 defined variants with possible regulatory effects. Using spectral, unsupervised learning algorithms, EIGEN [55] scores greater than 0 indicated putative deleterious variants. Additional annotations for intronic variants included Functional Analysis Through Hidden Markov Models-Multiple Kernel Learning (FATHMM-MLK) [56] scores greater than 0.50 and RegSNPs-intron [57] disease-causing probabilities greater than 0.50. LDproxy [58] was used to identify potentially functional QTNs in LD (r^2^ > 0.80) with QTNs significantly associated with MetS traits. These QTNs in LD were considered to be functional if they were exonic or had a RegulomeDB [59] rank (which ranged from 1 to 7) of at most 3.

## Results

### Descriptives

Sample and family size, demographic characteristics, and phenotypic measures varied by ethnic group (Table 1). There were 281 subjects in 73 AA families, 516 subjects in 75 EA families, 125 subjects in 15 JA families, and 598 subjects in 96 MA families. In particular, EA and JA families were larger and at least three-generational with a median size of 6–7 members per family, respectively. AA families were typically smaller with a median of 4 members per family. Although the MA families had a median family size of 4 members, the mean family size was 6 members, and there were a few very large MA families.

**Table 1:** GENNID genetic and phenotypic characteristics by ethnic group (mean ± SD)

Characteristics	AA: African Americans	EA: European Americans	JA: Japanese Americans	MA: Mexican Americans
# Subjects (# families)	281 (73 families)	516 (75 families)	125 (15 families)	598 (96 families)
Median (min, max) # Subjects per family	4 (1, 7)	6 (1, 29)	7 (3, 17)	4 (1, 112)
# QC^a^ variants	710,226	686,200	437,730	731,016
# Variants genotyped and imputed (association analysis)	13,042,663	7,681,619	5,455,666	7,907,815
Age (years)	52.4 ± 15.1	51.2 ± 16.8	56.1 ± 15.8	52.1 ± 15.8
Sex (% male)	31.3%	43.8%	52%	36.0%
Self-reported diabetes status (% diabetes)	51.2%	37.2%	27.20%	47.7%
Systolic blood pressure (SBP) (mmHg)	128.5 ± 19.8	127.2 ± 19.1	121.8 ± 18.1	126.2 ± 19.3
Diastolic blood pressure (DBP) (mmHg)	79.4 ± 10.8	78 ± 10.2	72.3 ± 11.5	74.6 ± 10.6
High density lipoproteins (HDL) (mg/dL)	47.5 ± 12.3	40.5 ± 11.2	45.6 ± 14.2	39.4 ± 10.2
Triglycerides (TG), fasting (mg/dL)	105.5 ± 110.3	143.8 ± 123.8	135.4 ± 104.5	160.9 ± 121.7
Glucose, fasting (mg/dL)	146.8 ± 75.3	128.3 ± 56.7	115 ± 29.6	152.2 ± 76.9
Insulin, fasting (mg/dL)	16.2 ± 21	11.5 ± 10.9	7.3 ± 6.3	17 ± 16.8
Weight (kg)	86.8 ± 21.1	84.1 ± 20.4	65.7 ± 13.4	79 ± 17.7
Waist circumference (cm)	98.5 ± 16	99.5 ± 16	88.7 ± 11.2	102.4 ± 14.3

*SD* standard deviation

^a^QC: quality control includes alignment to Haplotype Reference Consortium (HRC) panel (EA) or 1000 Genomes (1000G) panel (AA, MA, JA)

After QC, there was a similar number of QTNs for MA, AA, and EA families—about 731 K, 710 K, and 686 K QTNs, respectively. Among JA families, there were only ~ 437 K QTNs, which was ~ 40% less than the other ethnic groups. The lower number of QTNs among JA families was due to the removal of a large number of monomorphic markers, which may suggest a lower coverage for those of Asian descent on the multiethnic genotyping array [40].

Across ethnic groups, the mean age was similar and ranged between 51 and 56 years old (Table 1). About half the subjects in JA and EA families were men (52% and 43.8%, respectively), whereas AA and MA families had more females (68.7% and 64%, respectively). T2D was most frequent in AA and MA families with more than 51% and 47% of individuals self-reported as having T2D, respectively. About one-third of subjects (i.e., 37% and 27%) self-reported having T2D among EA and JA families, respectively. Although mean SBP and DBP measures were similar across ethnic groups, the mean blood pressure for this study population was elevated by 2017 clinical guidelines as defined by SBP > 120 mmHg and DBP > 80mmHg [60]. Furthermore, using NCEP ATP III guidelines [3], dyslipidemia, characterized by HDL measures < 40 mg/dL, was more evident in MA and EA families compared to AA and JA families (with the following means, respectively: 39.4 mg/dL and 40.5 mg/dL vs. 47.5 mg/dL and 45.6 mg/dL). Moreover, mean TG was most elevated among MAs with 160.9 mg/dL and lowest in AAs with 105.5 mg/dL. Hyperglycemia (when fasting glucose > 100 mg/dL) was present in all ethnic groups: MAs had the highest mean level (152.2 mg/dL) and JAs had the lowest mean level (115.0 mg/dL). Mean fasting insulin measures were elevated among MAs, AAs, and EAs (17.0 mg/dL, 16.2 mg/dL, and 16.5 mg/dL, respectively). Mean weight and mean waist circumference were both lowest among JAs (65.7 kg and 88.7 cm, respectively).

### Genomewide association results

**Fig. 1:**
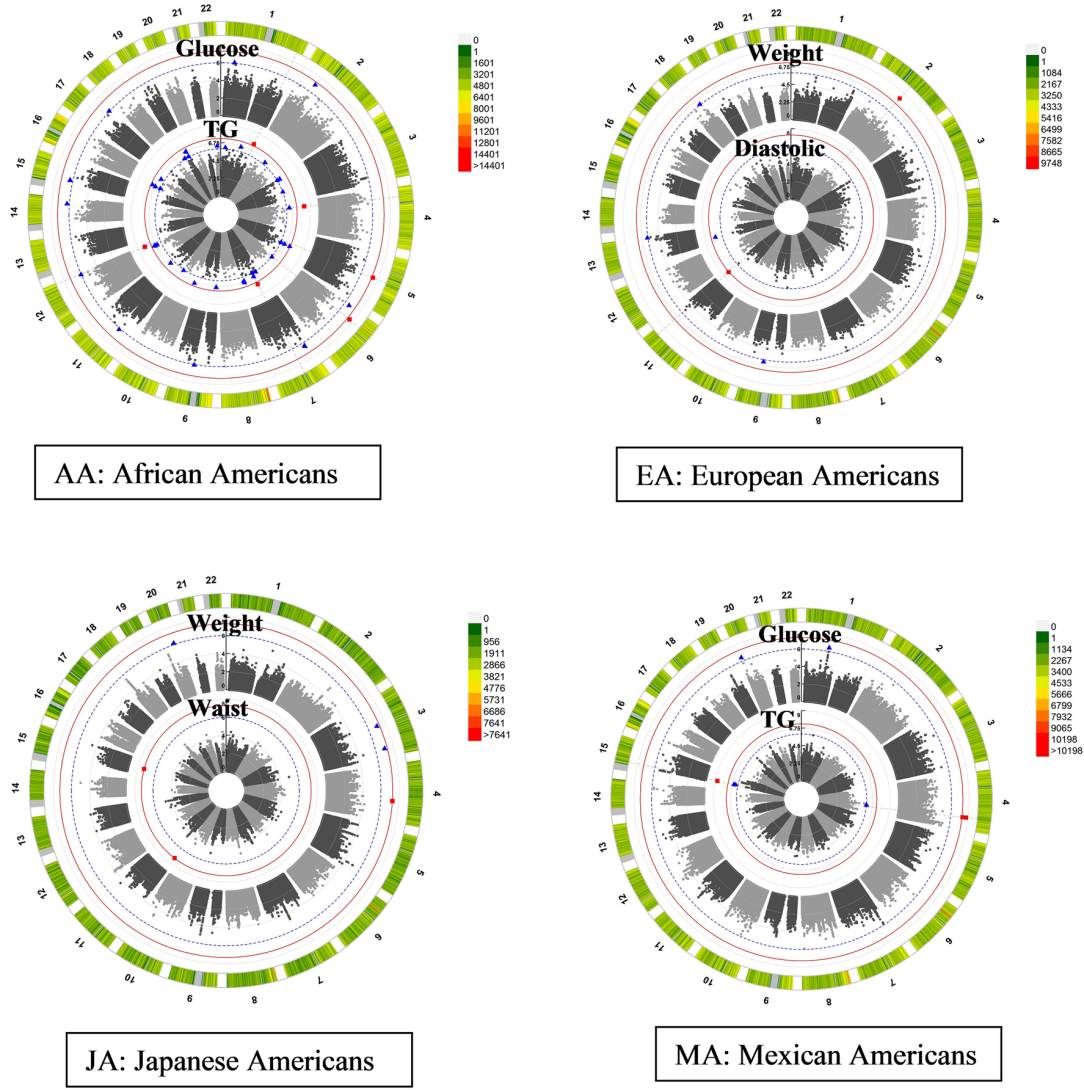
Family-based association results by Ethnic Group. Chromosomes are labeled 1 through 22 with QTN density denoted by right-hand legend. Each ring of the circular Manhattan plot indicates a selected quantitative trait that was analyzed (labeled at the top of each ring). Each dot represents the − log10(P) corresponding to test for association with the given quantitative trait. Genome-wide significant results are denoted by red box points at/above the red threshold line of − log10(5 × 10^−8^). Suggestive association is denoted by blue triangle points at/above the dotted blue line threshold at − log10(10^−6^). Only traits with significant results are shown for each ethnic group

We evaluated genetic association results for traits with at least one significant QTN (P ≤ 5 × 10^–8^) using circular Manhattan plots in Fig. 1. Table 2 presents all significant (P ≤ 5 × 10^–8^) results for each ethnic group including two variants with suggestive evidence (5 × 10^–8^ < P ≤ 10^–6^) of an association with MetS traits for EA: (a) rs1260326, a nonsynonymous *GCKR* variant with possible functional importance and (b) rs186742063, a possible pleiotropic variant with evidence of association with SBP and DBP traits. Specifically, there was a suggestive association of log(TG) and a non-synonymous QTN (rs1260326, P = 1.4 × 10^–7^) in the *GCKR* gene among the EAs. Additionally, in the EA group, on chromosome 11 at 115,495,297 bp (hg19/GRCh37) between *CADM1* and *LINC00900*, there was a pleiotropic QTN, rs186742063, with a significant association with DBP (P = 4.42 × 10^–8^) and a suggestive association with SBP (P = 9.92 × 10^–8^), respectively.

**Table 2:** Significant MetS Quantitative Trait Association results (P ≤ 5 × 10^–8^) by ethnic group

Ethnic group	Trait^a^	rsid	CHR	BP (hg19)	A1	A2	A1 Freq in study^b^	A1 Freq in AFR^c^	A1 Freq in AMR^c^	A1 Freq in EAS^c^	A1 Freq in NFE^c^
AA	log(TG)	rs75219957	1	231,296,755	G	C	0.020	0.012	0.133	0.278	0.0547
	log(TG)	rs568152609	4	31,060,113	C	T	0.010	0.007	0.002	0	0
	Glucose	rs138724790	5	86,997,218	C	T	0.010	0.016	0	0	0
	Glucose	rs78975723	6	39,338,990	G	C	0.010	0.033	0.002	0	6.5E−5
	log(TG)	rs73123056	7	63,354,705	A	C	0.040	0.024	0.073	0	0.048
	log(TG)	rs17137121	7	63,358,355	T	C	0.040	0.024	0.073	0	0.048
	log(TG)	rs114606502	12	130,117,352	C	T	0	0.007	0	0	0
	log(TG)	rs141776024	12	130,147,629	A	G	0	0.009	0	0	0
EA	log(TG)	rs1260326	2	27,730,940	T	C	0.400	0.135	0.314	0.501	0.400
	Weight	rs139940998	2	121,836,875	A	G	0.010	0.002	0.011	0	0.009
	Weight	rs144756634	2	121,843,978	A	T	0.010	0.002	0.011	0	0.009
	DBP	rs186742063^f^	11	115,495,297	A	G	0.020	0.004	0.007	0	0.018
JA	Weight	rs2302308	4	122,258,149	G	T	0.012	0.025	0.062	0.071	0.106
	Weight	rs74398478	4	122,259,584	A	C	0.012	0.025	0.063	0.071	0.106
	Weight	rs17051338	4	122,260,042	G	T	0.012	0.025	0.063	0.073	0.106
	Weight	rs77438622	4	122,264,692	G	C	0.012	0.025	0.063	0.073	0.106
	Weight	rs77006299	4	122,270,900	C	A	0.012	0.025	0.063	0.073	0.106
	log(waist)	rs146792726	11	3,972,232	A	G	0.012	0	0	0.029	0
	log(waist)	rs147605117	11	4,063,916	G	C	0.012	0	0	0.031	0
	log(waist)	rs143126598	15	54,259,502	A	G	0.012	0.0002	0	0.009	0
MA	Glucose	rs17024841	4	149,556,752	A	G	0.030	0.010	0.020	0.144	0.051
	Glucose	rs111933650	4	149,558,682	T	C	0.030	0.010	0.020	0.144	0.050
	Glucose	rs76209611	4	149,561,328	G	T	0.040	0.011	0.020	0.145	0.057
	log(TG)	rs4522365	15	29,964,742	T	C	0.170	0.534	0.156	0.088	0.152

Ethnic group	Trait^a^	rsid	Gene	Functional info	*I*^2d^	Beta	SE	P			
AA	log(TG)	rs75219957	FAM89A-TRIM67	Intergenic	80.4	1.04	0.19	3.0E−08			
	log(TG)	rs568152609	PCDH7	Intronic	–	1.36	0.23	5.3E−09			
	Glucose	rs138724790	CCNH-TMEM161B	Intergenic	–	1.93	0.35	3.7E−08			
	Glucose	rs78975723	KIF6	Intronic	–	1.75	0.31	2.6E−08			
	log(TG)	rs73123056	DQ599799-BC042811	Intergenic	84.5	0.81	0.15	2.4E−08			
	log(TG)	rs17137121	DQ599799-BC042811	Intergenic	84.5	0.81	0.15	2.4E−08			
	log(TG)	rs114606502	TMEM132D	Intronic	–	2.32	0.40	7.9E−09			
	log(TG)	rs141776024	TMEM132D	Intronic	–	2.32	0.40	7.9E−09			
EA	log(TG)	rs1260326	GCKR	Nonsyn	53.2	0.25	0.05	1.4E−07^e^			
	Weight	rs139940998	GLI2-TFCP2L1	Intergenic	–	1.84	0.32	7.3E−09			
	Weight	rs144756634	GLI2-TFCP2L1	Intergenic	–	1.84	0.32	7.3E−09			
	DBP	rs186742063^f^	CADM1-LINC00900	Intergenic	90.9	1.42	0.26	4.4E−08			
JA	Weight	rs2302308	QRFPR	Intronic	0	2.13	0.59	5.0E−08			
	Weight	rs74398478	QRFPR	Intronic	0	2.13	0.59	5.0E−08			
	Weight	rs17051338	QRFPR	Intronic	0	2.13	0.59	5.0E−08			
	Weight	rs77438622	QRFPR	Intronic	0	2.13	0.59	5.0E−08			
	Weight	rs77006299	QRFPR	Intronic	0	2.13	0.59	5.0E−08			
	log(waist)	rs146792726	STIM1	Intronic	–	− 0.39	0.08	5.0E−08			
	log(waist)	rs147605117	STIM1	Intronic	–	− 0.39	0.08	5.0E−08			
	log(waist)	rs143126598	WDR72-UNC13C	Intergenic	–	− 0.39	0.08	5.0E−08			
MA	Glucose	rs17024841	5S_rRNA-BC031092	Intergenic	80.6	− 0.70	0.12	1.7E−08			
	Glucose	rs111933650	5S_rRNA-BC031092	Intergenic	80.6	− 0.70	0.12	1.7E−08			
	Glucose	rs76209611	5S_rRNA-BC031092	Intergenic	78.9	− 0.67	0.12	4.2E−08			
	log(TG)	rs4522365	FAM189A1-BC043570	Intergenic	74.8	− 0.29	0.05	2.0E−09			

*AA* African Americans, *EA* European Americans, *JA* Japanese Americans, *MA* Mexican Americans

^a^Rank-based inverse normal transformation applied to weight, SBP (systolic blood pressure), DBP (diastolic blood pressure), and glucose traits

^b^Frequency of A1 allele in GENNID as estimated by GCTA [45]

^c^Frequency of A1 allele in gnomAD [76]: NFE (Non-Finnish Europeans) for EA, EAS (East Asians) for JA, AMR (Latinos) for MA, and AFR (African/African Americans) for AA

^d^Statistic summarizing heterogeneity across ethnic groups; the percentage of variation of beta estimates across ethnic groups due to heterogeneity; values of 0 indicate no heterogeneity of effect sizes across ethnic groups; values over 75% and up to 100% indicate high levels of heterogeneity; missing values indicate the QTN was only analyzed in one ethnic group (either due to the QTN being monomorphic in the other ethnic groups or being filtered out during quality control in the other ethnic groups)

^e^rs1260326 nonsynonymous SNV was suggestive (5 × 10^–8^ > P ≥ 10^–6^) but was found to be significant in GENNID meta-analysis [40]

^f^rs186742063 has evidence of pleiotropy with suggestive evidence (P = 9.9E−08) of being associated with systolic blood pressure (SBP) (see Additional file 1: Table S1)

Moreover, two variants were still significant after using a more stringent Bonferroni correction for analyzing 8 traits (i.e., P ≤ 6.25 × 10^–9^). Specifically, on chromosome 4, a rare, intronic *PCDH7* variant (rs568152609 with MAF = 0.7% in the AFR population of gnomAD) was strongly associated with log(TG) in AA. Additionally, in MA on chromosome 15, an intergenic variant (rs4522365) between *FAM189A1* and *BC043570* was also significantly associated with log(TG) (P = 2.0 × 10^–9^). All suggestive results (5 × 10^–8^ < P ≤ 10^–6^) are included in Additional file 1 (Table S1).

The significant QTNs associated with MetS quantitative traits are also from intergenic or intronic chromosomal regions. Most of the significant QTNs (Table 2) were moderately rare with ~ 1–3% frequency in the corresponding ethnic-specific population based on data from gnomAD reference groups [61]. These variants were enriched in our GENNID families and had frequencies (estimated by GCTA) that were ~ 1–2% higher than in gnomAD. QTNs significantly associated with log(waist) in JA and variants associated with glucose and log(TG) in MA all had negative additive effects (β < 0) for each copy of the A1 minor allele. All other significant associations had a positive additive effect (β > 0).

### Heterogeneity across ethnic groups

Although there were significant genetic associations with both fasting glucose and TG in both AA and MA (Fig. 1), the locations of the significant QTNs were found on different chromosomes (Table 2). For AA families, there were significant associations of log(TG) with multiple QTNs in the following genetic regions: 1q42.2, in *PCDH7* on chromosome 4, intergenic region between *DQ599799* and *BC042811* on chromosome 7, and in *TMEM132D* on chromosome 12. In addition, significant QTNs associated with fasting glucose levels were found in intergenic regions on chromosome 5 (*CCNH*-*TMEM161B*) and within the *KIF6* gene on chromosome 6. However, in MA families, log(TG) and fasting glucose levels were significantly associated with chromosomes 15q13.1 and 4q31, respectively. These regions were not associated with MetS quantitative traits in EA or JA groups (P > 10^–6^).

In addition, although in different physical locations, both EA and JA had significant genetic associations with weight (Fig. 1 and Table 2). For EA families, there was significant evidence of a genetic association for weight on chromosome 2q14.2, whereas in JA, QTNs were significantly associated with weight in the intronic region of *QRFPR* on chromosome 4. Moreover, in JA families, log-transformed waist circumference was significantly associated with QTNs on chromosomes 11 (*STIM1*) and 15 (at 54,259,502 bp between *WDR72* and *UNC13C*), whereas in EA there were only suggestive associations with log(waist) (Additional file 1: Table S1) on chromosome 2 between 123,052,152 and 124,754,047 bp (hg19/GRCh37) near *AX747402*, *7SK*, and *TSN*. These associations were unique to each ethnic group and were not found to be significant or suggestive in other ethnic groups (P > 10^–6^).

Thus, there was no overlap of significant or suggestive associations among ethnic groups. Moreover, there was evidence of heterogeneity between genetic effects across ethnic groups (*I*^2^ ≥ 75% in Table 2). Although chromosome 4 variants were significantly associated with weight only in JA families, these genetic effects were comparable across the other ethnic groups (i.e., *I*^2^ = 0). On the other hand, other variants besides the chromosome 4 *QRFPR* intronic variants associated with weight in JA families in Table 2, had effects that were either heterogeneous (*I*^2^ ≥ 75%) across ethnic groups or were unique to a single ethnic group (*I*^2^ not calculated) [61].

### Association in candidate linkage regions

**Fig. 2:**
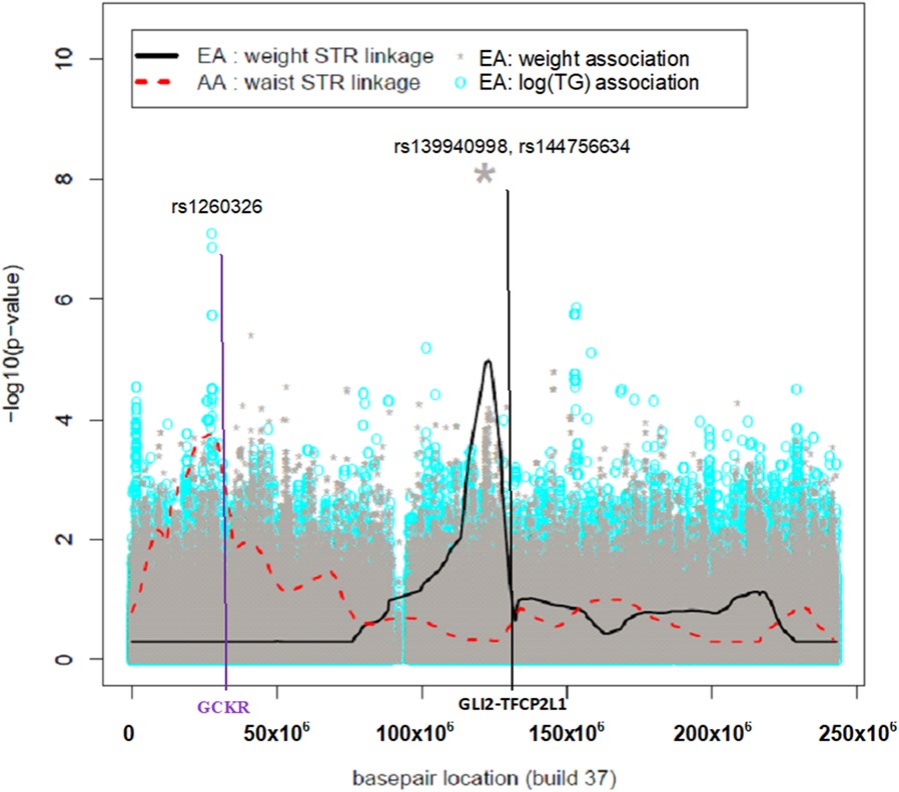
MetS Quantitative Trait Linkage and Association results: chromosome 2. Each color (black and red) and line type in the legend denotes ethnic group (European Americans (EA), African Americans (AA), respectively) univariate trait analysis for microsatellite (STR) linkage and SNP association testing. Genes of interest are denoted along the bottom of the x-axis in red and black font and colored vertical lines indicate suggestive and significant QTN locations. AA waist linkage had maxLOD = 2.78, and this location had prior evidence of association to GCKR

Some significant and suggestive associations overlapped or were near previously nominated candidate linkage regions found on chromosomes 1, 2, 3, 5, and 16 [32, 35, 36, 62–65] (Additional file 2: Table S2). In particular, unique only among EA families was a significant association between weight and QTNs (rs139940998 and rs144756634) on chromosome 2 at 121,836,875 bp and a second location at 121,843,978 bp (between *GLI2* and *TFC2L1*) within 7.3 Mbp of the candidate linkage region (2q12.1–13) (Fig. 2). Also, in the MA families, the candidate linkage region on 3p26 harbored two suggestive QTN associations (i.e., rs17005939 with P = 8.02 × 10^–8^ at chr3:2004251; rs12631510 with P = 9.13 × 10^–8^ at chr3:2001175) between the genes *CNTN6* and *CNTN4* at 3p26.3–p26.2 with log(HDL); both QTNs had genetic effects that were heterogeneous and differed across ethnic groups (*I*^2^ of 80.7% and 79.2%, respectively). Suggestive evidence for association with log(TG) and a non-coding RNA intron within a pseudo-gene, *AK126539*, (nominated by QTN at chr16:11562798) was identified within our linkage candidate region of 16p13.2–16p12.1 and was unique to AA families. Additional suggestive associations in AA with log(TG), specifically, three QTNs unique to AA and one QTN, rs78637841 in *WWOX*, having a high level of heterogeneity with *I*^2^ = 85%, and weight (i.e., three QTNs being unique to AA) were also found on 16q13.13 and 16q23.1, respectively (Additional file 1: Table S1). There was no evidence for association within the previously candidate linkage region on chromosome 5 (5q33.1–5q34) in JA (nominated for log(waist)); however, there was a nearby QTN (at chr5:130581195 within 5q23.3–5q31.1) that had suggestive association with fasting glucose (P = 1.38 × 10^–7^). The genetic effect of this QTN is not unique to JA and was comparable across ethnic groups (*I*^2^ = 0).

### Potential functional roles and regulatory effects

Additionally, in Table 3, we used ANNOVAR to assign functional roles to QTNs from Table 2 that were significantly associated with MetS traits. In particular, although the majority of significant QTNs were intronic and intergenic, some of these non-exonic variants have evidence of functionality according to the various annotating scoring methods. In the AAs, two intergenic QTNs (rs75219957 and rs73123056) on chromosomes 1 and 7, respectively, that were both significantly associated with log(TG) had evidence of possible deleterious effects. In the EAs, the *GCKR* nonsynonymous variant rs1260326 had moderate evidence of function based on an EIGEN score of 0.29, and the intergenic *GLI2-TFCP2L1* variant rs139940998 not only had a high CADD score of 19.05 but also evidence of possible deleterious effects from GWAVA, FATHMM, and EIGEN scores. Moreover, most of the significant *QRFPR* intronic variants associated with weight in JAs had moderate evidence of function based only on EIGEN scores; however, rs2302308 also had additional evidence of regulatory effects based on RegSNPs-intron with a 79% probability of being disease-causing. Also, in JAs, an *STIM1* intronic variant (rs147605117) significantly associated with waist circumference also had potential damaging effects as indicated by both a CADD score of 10.86 and EIGEN score of 0.29. On the other hand, in MAs, only one intergenic variant (rs76209611) had weak evidence of being functional with an EIGEN score near zero at 0.08.

**Table 3:** Significantly-associated MetS QTNs with functional annotation

Ethnic group	Trait	Gene (distance in basepairs) from refGene	Function (refGene)	CHR	Basepair (hg19)	rsid	Alleles	Functional annotations with thresholds^a^			
								FATHMM-MLK (noncoding > 0.50)	GWAVA (TSS) > 0.40	CADD v1.3 (phred-scaled) > 10	EIGEN > 0
AA	log(TG)	FAM89A(120,755) TRIM67(1919)	Intergenic	1	231,296,755	rs75219957	G/C	0.20	**0.62**	4.48	**0.38**
AA	log(TG)	MIR4283-1(273,158) ZNF722P(104,479)	Intergenic	7	63,354,705	rs73123056	A/C	**0.97**	0.21	3.38	**0.61**
EA	log(TG)	GCKR	Exonic-NSV^b^	2	27,730,940	rs1260326	T/C	0.02^c^	0.20	0.11	**0.29**
EA	Weight	GLI2(86,646) TFCP2L1(137,289)	Intergenic	2	121,836,875	rs139940998	A/G	**0.63**	**0.46**	**19.05**	**0.69**
JA	Weight	QRFPR	Intronic	4	122,258,149	rs2302308^d^	G/T	0.12	0.34	3.83	**0.02**
JA	Weight	QRFPR	Intronic	4	122,260,042	rs17051338	G/T	0.23	0.31	7.28	**0.33**
JA	Weight	QRFPR	Intronic	4	122,264,692	rs77438622	G/C	0.12	0.21	6.40	**0.09**
JA	Weight	QRFPR	Intronic	4	122,270,900	rs77006299	C/A	0.21	0.22	1.33	**0.03**
JA	Waist	STIM1	Intronic	11	3,972,232	rs146792726	A/G	0.14	0.12	4.48	**0.08**
JA	Waist	STIM1	Intronic	11	4,063,916	rs147605117	G/C	0.15	0.17	**10.86**	**0.28**
MA	Glucose	NR3C2(195,478) LOC105377480(300,770)	Intergenic	4	149,561,328	rs76209611	G/T	0.18	0.2	6.35	**0.08**

*AA* African Americans, EA, European Americans, JA, Japanese Americans, MA, Mexican Americans

^a^Bolded scores beyond recommended thresholds indicate QTNs with a deleterious or functional role

^b^Exonic, nonsynonymous variant

^c^FATHMM-MLK coding score shown

^d^RegSNPs-intron probability of disease-causing was 0.79 and only annotated for variant rs2302308

Furthermore, we determined if the significant QTNs located in intronic and intergenic regions were in LD with nearby, functional QTNs. Table 4 summarizes these proxy QTNs in LD with the significant QTN, with possible deleterious or regulatory effects given for each ethnicity. From the LDproxy [58] analysis, we found three QTNs that had high regulatory potential according to their RegulomeDB ranks; however, all of the proxy QTNs were in the same gene as the significant QTNs. Specifically, in the AAs, one *TMEM132D* intronic variant (rs14606502) was in LD with QTNs (rs142863227 and rs116163662) that had annotations of regulatory effects based on: transcription factor (TF) binding, having a DNase peak (indicating DNase I hypersensitivity sites), and/or being in any motif that could be functional. Notably, in the EAs, rs1260326 was in LD with another *GCKR* intronic variant, rs780094, that had evidence of regulatory effects including: TF binding, matched TF motif, and having a DNase peak. The five intronic *QRFPR* variants significantly associated with weight among the JAs were all in LD with rs55975435, an exonic but synonymous variant.

**Table 4:** Proxy QTNs in linkage disequilibrium (LD) with significantly-associated MetS QTNs

Ethnic group	Trait	QTN significantly associated with trait in Table 2		Proxy QTN (rsid)	CHR	bp (GRCh37)	Distance (bp)	MAF (%)^a^	r^2^ (LD)	Correlated alleles (significant QTN = proxy QTN)	RegulomeDB^b^	Gene: function
		Significant QTN (rsid)	Gene: function									
AA	log(TG)	rs114606502	TMEM132D: intronic	rs142863227	12	130,094,067	23,285	0.4	1	T = G C = C	4	TMEM132D: intronic
				rs116163662	12	130,114,869	2483	0.5	0.83	T = G C = A	3a	TMEM132D: intronic
EA	log(TG)	rs1260326	GCKR: exonic, Nonsynonymous	rs780094	2	27,741,237	10,297	41	0.91	T = T C = C	2c	GCKR: intronic
JA	Weight	rs2302308, rs74398478, rs17051338, rs77438622, rs77006299	QRFPR: intronic	rs55975435	4	122,254,014	6028	12	0.98	T = A G = G	7	QRFPR: exonic, synonymous

*AA* African Americans, *EA* European Americans, *JA* Japanese Americans, *MA* Mexican Americans

^a^Minor allele frequency (MAF) from 1000G corresponding reference group: AFR (for AA GENNID), EAS (for JA GENNID), EUR (for EA GENNID)

^b^RegulomeDB rank is defined by the following supporting data for evidence of regulatory effects: 2c: TF binding + matched TF motif + DNase peak; 3a: TF binding + any motif + DNase peak; 4: TF binding + DNase peak; 7: other; see https://www.regulomedb.org/regulome-help/

## Discussion

This study identified several suggestive and significant associations within previously defined candidate linkage regions. Multiple significant associations were also identified outside candidate regions, nominating other putative genes associated with MetS traits. We showed substantial heterogeneity as evidenced by trait-to-genotype associations that were unique to each ethnic group, a lack of sharing significant genetic associations between ethnic groups, and differences in genetic effects across ethnic groups. Interestingly, only one of these QTN associations (rs186742063) appeared to have pleiotropic effects only in the EA families. A large percentage of our findings were in intronic and intergenic regions, which are consistent with results of the ENCODE project [66].

There were several associations that were not within candidate linkage regions/regions. The only significant association findings on chromosome 1 was within 1q42.2, outside our candidate linkage region of 1q12–1q21.1 [32]. Among the EAs only and on chromosome 2, there were two intergenic *GLI2*-*TFCP2L1* variants (rs139940998 and rs144756634) that were associated with weight but did not lie within the previously identified candidate linkage region. In addition, the suggestively associated, non-synonymous QTN (rs1260326) within the *GCKR* gene in EA was found to be significant in both our trans-ethnic meta-analysis [40] as well as other studies [67, 68] and with evidence of nearby regulatory effects (Table 4). Moreover, the *GCKR* gene is located within a previous microsatellite linkage region nominated for harboring QTNs influencing the variation of waist circumference among GENNID AAs. Two suggestive associations on chromosome 3q26–27 region near the *CNTN4* gene were found to be unique to the MA sample. The *CNTN4* gene has been implicated with MetS traits [62, 63] and serum uric acid levels, and positively associated with increased risk for chronic kidney disease and cardiovascular disease [65]. The *ADIPOQ* gene was nominated as a candidate gene in our linkage region on 3p26 [32]; however, in the current study there was no evidence of associated QTNs in the *ADIPOQ* region with any of the univariate MetS traits, despite previous reports of ADIPOQ being associated with multiple underlying MetS conditions [8] and being associated with MetS in Han Chinese [69] and North Indian Punjabi [70]. In the GENNID JA families, there were two intronic *STIM1* variants (rs146792726 and rs147605117) on chromosome 11 that were significantly associated with waist circumference (with the latter variant having evidence of function based on CADD and EIGEN scores). However, both variants were in linkage equilibrium (uncorrelated) with another *STIM1* variant rs12290747 (r^2^ = 0.004) that was found to be significantly associated with urolithiasis in a recent, large-scale Japanese population GWAS [71].

Together the linkage and association results indicated differences in genetic and phenotypic architecture that are unique to each ethnic group. Furthermore, there was no overlap in the significant genes nominated among the four ethnicities. For example, we found significant evidence of a genetic association on chromosome 2q14.2 with weight and waist circumference in EAs; these two QTNs (rs139940998, rs144756634) are located in between *GLI2* and *TFCP2L1*, which is expressed in the kidneys [72] and may act as a transcriptional suppressor of UBP1-mediated transcription activation [73]. Moreover, rs139940998 was identified as being functional across multiple annotations (Table 3). However, among JA, weight was significantly associated with the intronic region of *QRFPR* on chromosome 4, and there were associations between waist circumference and QTNs on chromosomes 11 (*STIM1*) and 15 (between *WDR72* and *UNC13C*). These two regions were not nominated in the MA and AA groups. Similarly, in MA families, a significant genetic association was demonstrated between log(TG) and 15q13.1, but in AA families, log(TG) was significantly associated with multiple QTNs in 1q42.2, *PCDH7* on chromosome 4, the intergenic region between *DG599799* and *BC04811* on chromosome 7, and in *TMEM132D* with possible regulatory effects (Table 4) on chromosome 12. These findings are consistent with our previous studies [32, 33] which have shown that the clustering of MetS traits in the GENNID differs by ethnicity. The Multi-Ethnic Study of Atherosclerosis (MESA), also found heterogeneity of effects across ethnic groups and ethnic-specific results while investigating genetic associations of body mass index (BMI); in particular the intronic rs6435678 in *ERBB4* on chromosome 2 was significantly associated with BMI only in AA; however, these results were uncorrelated with our significant chromosome 2 associations with weight in the GENNID EA families [74]. This phenotypic heterogeneity could be driven by different sets of underlying genes [34] that could in turn explain variation in risk for MetS-related conditions. LD analysis and annotation of our top candidate QTNs revealed possible regulatory roles in several of these genes. However, additional functional validation studies are needed.

Furthermore, the use of family-based analyses enabled us to use a sample size smaller than what is needed for a traditional case–control GWAS to detect rare QTN associations [75]. Although the JAs had the fewest number of families, these families were multi-generational (at least three generations deep) and had more members per family. Nonetheless, for the JAs, we used genedropping to calculate p-values using empirical distributions for statistics when large-sample, asymptotic approximations may not have been valid. In addition, we used conservative genome-wide significance thresholds to assess associations in our candidate linkage regions. However, we may have been too conservative in our univariate association testing approach. Further multivariate trait analyses accounting for between trait correlations could increase power to detect genetic associations and pleiotropy.

## Conclusions

Our study associations of MetS traits across a diverse group of multiethnic Americans. We built on our previous linkage analysis using highly informative microsatellites and utilized the power of genome-wide QTN association testing in families to refine and extend our previous work examining evidence for heterogeneity and pleiotropy. In summary, heterogeneity across ethnic groups was evident in not only the genetic location of the QTN, but with different associated traits and genetic effects. There was some evidence of intergenic and intronic variants having functional properties based on annotation scoring. Most significant associations were outside our candidate linkage regions and were coincident. However, in EA families and within the chromosome 2 candidate region, two significant *GLI2-TFCP2L1* associations with weight were found; also, there was one chromosome 11 variant (rs186742063) with pleiotropic blood pressure effects found in the EAs. The results of this project provide new insights into the complexity and genetic architecture of MetS and highlight the utility of family-based studies and the importance of including diverse populations in genomic research.

## Supplementary Information

**Additional file 1.** **Table S1**: Suggestive MetS association results (5 × 10^−8^ < p < 10^−6^) by ethnic group. Summary Table of suggestive results.

**Additional file 2.** **Table S2**: Association within or nearby linkage region^a^ (P< 10^−6^ and LOD ≤ 1.9). Summary Table of association results within or nearby candidate linkage regions.

## Data Availability

The data that support the findings of this study are available from American Diabetes Association but restrictions apply to the availability of these data, which were used under license for the current study, and so are not publicly available. Data are however available from the authors upon reasonable request and with permission of American Diabetes Association.

## Abbreviations

GENNID: GENetics of Noninsulin dependent Diabetes Mellitus
MetS: Metabolic syndrome
NCEP ATP III: National Cholesterol Education Program’s Adult Treatment Panel III
HDL: High-density lipoproteins
TG: Triglycerides
SBP: Systolic blood pressure
DBP: Diastolic blood pressure
GWAS: Genome-wide association studies
EA: European American
MA: Mexican American
AA: African American
JA: Japanese American
T2D: Type 2 diabetes
QTN: Quantitative trait nucleotide
ADA: American Diabetes Association
NWGC: Northwest Genomics Center
QC: Quality control
HRC: Haplotype Reference Consortium
GCTA: Genome-wide Complex Trait Analysis
LDAK: LD-adjusted kinships
LD: Linkage disequilibrium
ANNOVAR: ANNOtate VARiation
SVM: Support vector machine
CADD: Combined Annotation Dependent Depletion
GWAVA: Genome Wide Annotation of VAriants
FATHMM-MLK: Functional Analysis Through Hidden Markov Models-Multiple Kernel Learning
MESA: Multi-Ethnic Study of Atherosclerosis
