# Supplementary material for: Genome-wide association analysis of metabolic syndrome quantitative traits in the GENNID multiethnic family study
Source: Diabetol Metab Syndr. 2021 Jun 1;13:59. doi: 10.1186/s13098-021-00670-3 (PMC8170963; doi:10.1186/s13098-021-00670-3)
Supplement: Supplementary file 2 — Additional file 2. Table S2: Association within or nearby linkage regiona (P< 10−6 and LOD ≤ 1.9). Summary Table of association results within or nearby candidate linkage regions. [file 13098_2021_670_MOESM2_ESM.pdf]

Additional file 2. Association within or nearby linkage region[a] (P<10^{-6} & LOD≤1.9)

	Ethnic Group	EA: European Americans		AA: African Americans	MA: Mexican Americans		JA: Japanese Americans
**PUBLISHED LINKAGE RESULTS**	**1-LOD chromosomal region (Trait & LOD of linkage analysis)**	**2q12.1-2q13 (Weight, LOD=2.91)**		**16p13.2-12.1 (BPfactor, LOD=2.25)**	**3p26 (Insulin-Glucose, Blood Pressure factors, LOD=2.20)(1) (Serum uric acid, LOD=4.9)(2)**		**5q33.1-5q34 (log-Waist, LOD=1.98)**
**ASSOCIATION RESULTS**	**trait**	Weight	Weight	log(TG)	log(HDL)	log(HDL)	Fasting Glucose
	**Chromosomal Region**	2q14.2		16p13.13	3p26.3-p26.2		5q23.3-5q31.1[b]
	**Chr**	2	2	16	3	3	5
	**Bp**	121836875	121843978	11562798	2004251	2001175	130581195
	**rsid**	rs139940998[c]	rs144756634[c]	rs551107164	rs17005939	rs12631510	rs6875363
	**Gene**	GLI2-TFCP2L1		AK126539	CNTN6-CNTN4		LYRM7-CDC42SE2
	**Function**	intergenic	intergenic	ncRNA_intronic	intergenic	intergenic	intergenic
	**A1**	A	A	G	C	C	T
	**A2**	G	T	A	T	T	C
	**Freq[§]**	0.0087	0.0089	0.011	0.4186	0.4375	0.6857
	**Freq GENNID[‖]**	0.01	0.01	0.01	0.35	0.35	0.4421
	**β**	1.84	1.84	1.41	-0.08	-0.08	-0.54263
	**SE**	0.32	0.32	0.26	0.02	0.02	0.109372
	**P**	7.34E-09	7.34E-09	7.61E-08	8.02E-08	9.13E-08	1.38E-07
	**I^2 (% heterogeneity)**	-	-	-	80.7%	79.2%	0%[h]

[a]Candidate linkage regions for EA, AA and JA were from publication analyses (3-7)

[b]Region with evidence of association is very close to previously nominated 1-LOD interval but not within it

[c]Bolded variants were significantly associated with weight (P<5x10^{-8}).

[f]Frequency of A1 allele in gnomAD (8) : NFE (Non-Finnish Europeans) for EA, EAS (East Asians) for JA, AMR (Latinos) for MA, and AFR (African/African Americans) for AA

[g] Frequency of A1 allele in GENNID as estimated by GCTA (9)

[h] I^2 = -51.6% and was to set 0% based on Higgins et al **(**10**)**

REFERENCES FOR ADDITIONAL FILE

1. Cai G, Cole SA, Freeland-Graves JH, MacCluer JW, Blangero J, Comuzzie AG: Principal component for metabolic syndrome risk maps to chromosome 4p in Mexican Americans: the San Antonio Family Heart Study. Hum Biol 2004;76:651-665
2. Chittoor G, Kent JW, Jr., Almeida M, Puppala S, Farook VS, Cole SA, Haack K, Goring HH, MacCluer JW, Curran JE, Carless MA, Johnson MP, Moses EK, Almasy L, Mahaney MC, Lehman DM, Duggirala R, Comuzzie AG, Blangero J, Voruganti VS: GWAS and transcriptional analysis prioritize ITPR1 and CNTN4 for a serum uric acid 3p26 QTL in Mexican Americans. BMC Genomics 2016;17:276
3. Edwards KL, Hutter CM, Wan JY, Kim H, Monks SA: Genome-wide linkage scan for the metabolic syndrome: the GENNID study. Obesity (Silver Spring) 2008;16:1596-1601
4. Elbein SC, Das SK, Hallman DM, Hanis CL, Hasstedt SJ: Genome-wide linkage and admixture mapping of type 2 diabetes in African American families from the American Diabetes Association GENNID (Genetics of NIDDM) Study Cohort. Diabetes 2009;58:268-274
5. Hasstedt SJ, Highland HM, Elbein SC, Hanis CL, Das SK, American Diabetes Association GSG: Five linkage regions each harbor multiple type 2 diabetes genes in the African American subset of the GENNID Study. J Hum Genet 2013;58:378-383
6. Kissebah AH, Sonnenberg GE, Myklebust J, Goldstein M, Broman K, James RG, Marks JA, Krakower GR, Jacob HJ, Weber J, Martin L, Blangero J, Comuzzie AG: Quantitative trait loci on chromosomes 3 and 17 influence phenotypes of the metabolic syndrome. Proc Natl Acad Sci U S A 2000;97:14478-14483
7. Rich SS, Bowden DW, Haffner SM, Norris JM, Saad MF, Mitchell BD, Rotter JI, Langefeld CD, Wagenknecht LE, Bergman RN, Insulin Resistance Atherosclerosis Study Family S: Identification of quantitative trait loci for glucose homeostasis: the Insulin Resistance Atherosclerosis Study (IRAS) Family Study. Diabetes 2004;53:1866-1875
8. Karczewski KJ, Francioli LC, Tiao G, Cummings BB, Alföldi J, Wang Q, Collins RL, Laricchia KM, Ganna A, Birnbaum DP, Gauthier LD, Brand H, Solomonson M, Watts NA, Rhodes D, Singer-Berk M, England EM, Seaby EG, Kosmicki JA, Walters RK, Tashman K, Farjoun Y, Banks E, Poterba T, Wang A, Seed C, Whiffin N, Chong JX, Samocha KE, Pierce-Hoffman E, Zappala Z, O'Donnell-Luria AH, Minikel EV, Weisburd B, Lek M, Ware JS, Vittal C, Armean IM, Bergelson L, Cibulskis K, Connolly KM, Covarrubias M, Donnelly S, Ferriera S, Gabriel S, Gentry J, Gupta N, Jeandet T, Kaplan D, Llanwarne C, Munshi R, Novod S, Petrillo N, Roazen D, Ruano-Rubio V, Saltzman A, Schleicher M, Soto J, Tibbetts K, Tolonen C, Wade G, Talkowski ME, Neale BM, Daly MJ, MacArthur DG: Variation across 141,456 human exomes and genomes reveals the spectrum of loss-of-function intolerance across human protein-coding genes. bioRxiv 2019:531210
9. Yang JA, Lee SH, Goddard ME, Visscher PM: GCTA: A Tool for Genome-wide Complex Trait Analysis. American Journal of Human Genetics 2011;88:76-82
10. Higgins JP, Thompson SG, Deeks JJ, Altman DG: Measuring inconsistency in meta-analyses. BMJ 2003;327:557-560
